# Functional Analysis of the Endopeptidase and Holin From *Planktothrix agardhii* Cyanophage PaV-LD

**DOI:** 10.3389/fmicb.2022.849492

**Published:** 2022-04-28

**Authors:** Li-Hui Meng, Fei Ke, Qi-Ya Zhang, Zhe Zhao

**Affiliations:** ^1^Department of Marine Biology, College of Oceanography, Hohai University, Nanjing, China; ^2^State Key Laboratory of Freshwater Ecology and Biotechnology, Institute of Hydrobiology, Chinese Academy of Sciences, Wuhan, China

**Keywords:** *Planktothrix agardhii*, cyanophage PaV-LD, endopeptidase, holin, *Synechocystis* sp. PCC6803

## Abstract

A cyanophage PaV-LD, previously isolated from harmful filamentous cyanobacterium *Planktothrix agardhii*, was sequenced, and co-expression of its two ORFs in tandem, ORF123 and ORF124, inhibited growth on the model cyanobacterium *Synechocystis* sp. PCC6803 cells. However, the mechanism of action of ORF123 and ORF124 alone remains to be elucidated. In this study, we aimed to study the individual function of ORF123 or ORF124 from PaV-LD. Our data showed that the ORF123 encoded an endopeptidase, which harbored an M23 family peptidase domain and a transmembrane region. The expression of the endopeptidase in *Escherichia coli* alone revealed that the protein exhibited remarkable bacteriostatic activity, as evidenced by observation of growth inhibition, membrane damage, and leakage of the intracellular enzyme. Similarly, the holin, a membrane-associated protein encoded by the ORF124, showed weak bacteriostatic activity on *E. coli*. Moreover, deletion mutations indicated that the transmembrane domains of endopeptidase and holin were indispensable for their bacteriostatic activity. Meanwhile, the bacteriostatic functions of endopeptidase and holin on cyanobacteria cells were confirmed by expressing them in the cyanobacterium *Synechocystis* sp. PCC6803. Collectively, our study revealed the individual role of endopeptidase or holin and their synergistic bacteriolytic effect, which would contribute to a better understanding of the lytic mechanism of cyanophage PaV-LD.

## Introduction

Cyanobacteria, which have existed on the earth for more than 3.5 billion years, are widely distributed in aquatic environments (Altermann et al., 2006; Beraldi-Campesi, 2013; Paerl and Otten, 2013). As a dominant group of photosynthetic bacteria in most aquatic environments, cyanobacteria provide the source of primary production of oxygen, nitrogen, and carbon, and act as a model organism for studying the coordination of carbon and nitrogen metabolisms (Jiang et al., 2017). Cyanophages, a group of viruses that specifically infect cyanobacteria, are key factors that mediate the host communities, food web, carbon cycling, and nutrient recycling (Yang et al., 2020). They also have a potential impact on the regulation of cyanobacterial bloom through lysis-induced mortality, metabolic outputs, and altering diversity and community structures (Zhang and Gui, 2018). In recent decades, with the increasing frequency of water blooms, more and more attention has been paid to the infection and killing of cyanobacteria by cyanophages. It is particularly necessary to clarify the mechanism of host lysis by cyanophages, which will provide the theoretical basis for finding alternative strategies to control cyanobacteria biomass.

Endolysin and holin homologs are commonly found in cyanophages and are believed to have a lytic function on host cells (Yoshida et al., 2007; Chénard et al., 2015; Zhong et al., 2018). Endolysins are phage-encoded enzymatic proteins that are synthesized at the end stage of phage replication inside the hosts (Bai et al., 2020). According to the target sites of the bacterial peptidoglycan, endolysins could be divided into five categories: glucosidase, muramidase, amidase, endopeptidase, and transglycosylase (Gondil et al., 2019; Nl et al., 2020). They can hydrolyze bacterial peptidoglycan to degrade the cell wall and then release new phage particles from host cells (Schuch et al., 2002). Holins, another essential component for host lysis, are a kind of hydrophobic membrane protein usually encoded by double-strand DNA phages (Wang et al., 2000; Reddy and Saier, 2013). They cause non-specific pores or lesions in the cytoplasmic membrane of hosts at the late stage of infection to allow the endolysins to reach the cell wall compartment and perform effectively (Savva et al., 2014). Although holins vary in size from 49 amino acid (aa) to 210 aa, they do have some common characteristics (Wang et al., 2000) as follows: (1) the genes encoding most holins are adjacent to the endolysin genes; (2) holins possess at least one transmembrane α-helical sequence (Reddy and Saier, 2013); and (3) holins have a highly charged, hydrophilic, C-terminal domain. In addition, holins could generally be grouped into three classes by topology according to the length of the aa and the number of the transmembrane domain (TMD) (Class I: length more than 95 aa with 3 TMDs; Class II: length of 65–95 aa with 2 TMDs; Class III: containing 1 TMD) (Wang et al., 2000; Reddy and Saier, 2013). Recent reports showed that the holin-endolysin of *Salmonella* phage P22 can inhibit the growth of cyanobacteria (Liu and Roy, 2009; Zhou et al., 2019).

To date, lots of endolysins and holins from phages have been studied as antibacterial compounds, such as *Staphylococcus aureus* bacteriophage GH15, *Salmonella* phage P22, and *Streptomyces avermitilis* bacteriophage phiSASD1 (Song et al., 2016; Zhou et al., 2019; Nl et al., 2020). However, few studies focus on the homologous proteins from cyanophage. In our previous study, a *Planktothrix agardhii* cyanophage PaV-LD was isolated and sequenced (Gao et al., 2012). The genome annotation revealed that a putative lysis module (ORF123 and ORF124 encoding endopeptidase and holin, respectively) existed in the cyanophage genome (Gao et al., 2012). Subsequently, co-expression of endopeptidase and holin showed the inhibition effect on the growth of PCC6803 cyanobacteria cells, indicating they probably operate to lyse host cells (Li et al., 2013). In this study, the individual functions of endopeptidase and holin were further investigated based on *Escherichia coli* and *Synechocystis* sp. PCC6803 expression system. These data would help to reveal their basic biological functions and better understand the lytic mechanism of PaV-LD.

## Materials and Methods

### Bioinformatic Analysis

The amino acid sequences of endopeptidase and holin from the cyanophage PaV-LD were used as queries to search the non-redundant database using BLASTP. A domain search was performed against the Pfam database and the NCBI’s Conserved Domain Database. TMHMM^1^ was used to predict the transmembrane helices of endopeptidase and holin.

### Bacteria, Cyanobacteria, and Cyanophage Culture

Bacterial strains used in this study were listed in Table 1. *Escherichia coli* DH5α was used for cloning experiments and plasmid isolation, and *E. coli* BL21 (DE3) was used to produce recombinant proteins. All bacterial strains were cultivated in Luria–Bertani (LB) broth or agar at 37°C. Unless otherwise indicated, the concentration of ampicillin (Amp) was added to the medium with 100 μg/ml. *Planktothrix agardhii* HAB0637 and *Synechocystis* sp. PCC6803 were grown in liquid medium BG11, in a light incubator under a light:dark cycle of 12:12 h with a constant illumination of ∼30 μE⋅m^–2^s^–1^ at 25°C. The cyanophage PaV-LD was propagated using the host strain *P. agardhii* HAB0637 as described previously (Gao et al., 2012).

**TABLE 1:** Strains/cyanophages used in this study.

Strains/cyanophages	Genotype or relevant properties	Source
*Escherichia coli* DH5α	*supE*44, Δ*lacU*169 (φ80 *lacZ*ΔM15), *hsdR*17, *recA*, *endA*1, *gyrA*96, *thi-1*, and *relA*1	Takara
*Escherichia coli* BL21 (DE3)	*E. coli B, F-, ompT, hsdSB, (rB-, mB-), dcm, gal*, and *k(DE3) pRARE (CamR)*	ZOMANBIO
DE3: pET-21a	Strains containing plasmid pET-21a	This lab
DE3: pET-21a-endopeptidase	Strains containing plasmid pET-21a-endopeptidase	This lab
DE3: pET-21a-holin	Strains containing plasmid pET-21a-holin	This lab
DE3: pET-32a	Strains containing plasmid pET-32a	This lab
DE3: pET-32a-endopeptidase	Strains containing plasmid pET-32a-endopeptidase	This lab
DE3: pET-32a-holin	Strains containing plasmid pET-32a-holin	This lab
DE3: pET-32a-ORF59	Strains containing plasmid pET-32a-ORF59	This lab
DE3: pET-21a-endopeptidaseΔTM	Strains containing plasmid pET-21a-endopeptidaseΔTM	This lab
DE3: pET-21a-holinΔTM	Strains containing plasmid pET-21a-holinΔTM	This lab
DE3: pET-32a*-*GFP	Strains containing plasmid pET-32a*-*GFP	This lab
DE3: pET-32a-endopeptidase*-*GFP	Strains containing plasmid pET-32a-endopeptidase*-*GFP	This lab
DE3: pET-32a-holin*-*GFP	Strains containing plasmid pET-32a-holin*-*GFP	This lab
DE3: pETDuet-1	Strains containing plasmid pETDuet-1	This lab
DE3: pETDuet-endopeptidase	Strains containing plasmid pETDuet-endopeptidase	This lab
DE3: pETDuet-holin	Strains containing plasmid pETDuet-holin	This lab
DE3: pETDuet-holin-endopeptidase	Strains containing plasmid DE3: pETDuet-holin-endopeptidase	This lab
*Synechocystis* sp. PCC6803	model cyanobacterium	This lab
PCC6803: pHB-endopeptidase	Strains containing plasmid pHB-endopeptidase	This lab
PCC6803: pHB-holin	Strains containing plasmid pHB-holin	This lab
*Planktothrix agardhii* HAB0637	Host for cyanophage PaV-LD	This lab
Cyanophage PaV-LD	Wild-type, 95299bp dsDNA genome	This lab

### Plasmid Construction

Genomic DNA of cyanophage PaV-LD was used as a template for polymerase chain reaction (PCR) with corresponding primers (Table 2), using high-fidelity DNA polymerase (Vazyme, Nanjing, China). PCR products containing the ORF123 and ORF124 were purified using a PCR Purification Kit (OMEGA, Norcross, GA, United States), and the target segments were ligated into the expression vectors pET-21a (+) and pET-32a (+) using a One Step Cloning Kit (Vazyme, Nanjing, China), respectively. The constructed plasmids were transformed into competent *E. coli* BL21 (DE3) for expression, respectively. The recombinant plasmids pET-21a-endopeptidaseΔTM and pET-21a-holinΔTM were constructed using the primers ORF123ΔTM-FW/RV and ORF124ΔTM-FW/RV as described above. In addition, we constructed the fusion expression plasmids pET-32a-endopeptidase-GFP and pET-32a-holin-GFP to observe the cellular localization of endopeptidase and holin in *E. coli.* To explore whether there is synergy between endopeptidase and holin, we constructed a double expression recombinant plasmid with the pETDuet-1 vector, and the resulting plasmid was designed as pETDuet-holin-endopeptidase. The pHB-Con plasmid kept in our laboratory was used to construct the recombinant plasmids pHB-endopeptidase and pHB-holin using the primers pHB-ORF123-FW/RV and pHB-ORF124-FW/RV, respectively. The pHB-Con (*slr0168*C-*Omega*-*petE*-*slr0168*N) contained the *rbcL* gene of PCC6803 cyanobacteria, and the spectinomycin resistance gene omega and promoter *petE* were inserted into the gene. The plasmid could be used to construct a transformation plasmid expressing foreign genes in PCC6803 cyanobacteria. After the transfected plasmid entered the cyanobacteria cells, the carried nucleic acid fragment would be integrated into the *rbcL* gene of the PCC6803 genome by homologous recombination, and the expression of the target gene was induced by regulating the promoter. All cloned fragments were verified *via* sequencing analysis.

**TABLE 2:** Primers used in this study.

Primer sequence (5′–3′)	
pET-21a-ORF123-FW	atgggtcgcggatccgaattcATGAATCAGAAACTGGAGGAGAGG
pET-21a-ORF123-RV	ttgtcgacggagctcgaattc TTTTTTTGGCTCCATTATAGGGA
pET-21a-ORF124-FW	atgggtcgcggatccgaattc ATGAGAGTTATTTTTTATGCTGGAG
pET-21a-ORF124-RV	ttgtcgacggagctcgaattc TTTGACTCCCATATCCTGTTTC
pET-32a-ORF124-FW	gctgatatcggatccgaattc ATGAGAGTTATTTTTTATGCTGGAG
pET-32a-ORF124-RV	ttgtcgacggagctcgaattc TTTGACTCCCATATCCTGTTTC
pET-32a-ORF123-FW	gctgatatcggatccgaattc ATGAATCAGAAACTGGAGGAGAGG
pET-32a-ORF123-RV	ttgtcgacggagctcgaattc TTTTTTTGGCTCCATTATAGGGA
pET-32a-ORF59-FW	gctgatatcggatccgaattcATG GTCGGCAAAGGTCTCC
pET-32a-ORF59-RV	ttgtcgacggagctcgaattc CGCCCGGGCAGGTGTA
pETDuet-ORF123-FW	gctgacgtcggtaccctcgag ATGAATCAGAAACTGGAGGAGAGG
pETDuet-ORF123-RV	ggtttctttaccagactcgag TTTTTTTGGCTCCATTATAGGGA
pETDuet-ORF124-FW	tcatcaccacagccaggatcc ATGAGAGTTATTTTTTATGCTGGAGG
pETDuet-ORF124-RV	gccgagctcgaattcggatcCCTATTTGACTCCCATATCCTGTTTCC
pET-21a-ORF123ΔTM-FW	atgggtcgcggatccgaattc ATGCCCTCTCCCCAGGTTC
pET-21a-ORF123ΔTM-RV	ttgtcgacggagctcgaattc TTTTTTTGGCTCCATTATAGGGA
pET-21a-ORF124ΔTM-FW	atgggtcgcggatccgaattcATGAAGAAAGGGTTTGCTGAA
pET-21a-ORF124ΔTM-RV	ttgtcgacggagctcgaattc TTTGACTCCCATATCCTGTTTC
pET-32a-GFP-*Not*I-FW	tccgtcgacaagcttgcggccgcATGGTGAGCAAGGGCGAGGA
pET-32a-GFP-*Not*I-RV	tggtggtgctcgagtgcggccgcCTTGTACAGCTCGTCCATGCCG
pHB-FW	CCCATATAACCATCAAAGCCATAG
pHB-RV	ATCTCTAGAGGAGCTTGCATGCC
ORF123-FW	ATGAATCAGAAACTGGAGGAGAGG
ORF123-RV	TCATTTTTTTGGCTCCATTATAGG
ORF124-FW	ATGAGAGTTATTTTTTATGCTGGAGG
ORF124-RV	CTATTTGACTCCCATATCCTGTTTCC
pHB-ORF123-FW	atgcaagctcctctagagatATGAATCAGAAACTGGAGGAGAGG
pHB-ORF123-RV	ggctttgatggttatatgggTCATTTTTTTGGCTCCATTATAGG
pHB-ORF124-FW	atgcaagctcctctagagatATGAGAGTTATTTTTTATGCTGGAGG
pHB-ORF124-RV	ggctttgatggttatatgggCTATTTGACTCCCATATCCTGTTTCC

*FW, sense primer; RV, antisense primer.*

### Localization of Endopeptidase and Holin in *Escherichia coli*

These strains (DE3: pET32a-endopeptidase-GFP, DE3: pET32a-holin-GFP, and DE3: pET32a-GFP) were grown in LB broth Amp. When the OD_600_ of the culture reached 0.6, isopropyl-β-D-thiogalactoside (IPTG) was added to a final concentration of 0.1 mM, and the growth temperature was adjusted to 30°C. After 4 h of induction, the cells were harvested by centrifugation at 10,000 rpm for 10 min, washed with PBS, and observed by fluorescence microscope.

To further confirm the cell localization of endopeptidase and holin in *E. coli*, different cell components were isolated from *E. coli* (DE3: pET32a, DE3: pET32a-ORF59, DE3: pET32a-endopeptidase, and DE3: pET32a-holin) for Western blotting. The membrane-associated fractions were isolated using an *E. coli* membrane protein extraction kit (BestBio, Shanghai, China) according to the manufacturer’s instructions. The whole-cell, cytoplasmic fraction and membrane fraction extracts were, respectively, examined by Western blotting. Briefly, after electrophoresis, the proteins were directly transferred from the gel onto a PVDF membrane (Millipore, Burlington, MA, United States) by electroblotting. For the immunodetection of holin, anti-His-tagged mAb (CST, Trask Lane Danvers, MA, United States) and HRP-labeled goat anti-mouse IgG (Beyotime, Shanghai, China) were used as the primary and secondary antibodies, respectively. The bands were detected with Pierce ECL Plus Western Blotting Substrate (Thermo Fisher Scientific, Waltham, MA, United States). In this experiment, empty pET32a and pET32a-ORF59 plasmids were used as control groups. Among them, ORF59 was a membrane protein previously identified by our team (Li et al., 2021).

### Bacterial Growth Curve Assays

To examine the effects of endopeptidase, holin, and their TMD deletion mutants on the growth of *E. coli*, different plasmids, including pET-21a-endopeptidase, pET-21a-holin, pET-21a-endopeptidaseΔTM, and pET-21a-holinΔTM, were transformed into competent *E. coli* BL21 (DE3), respectively. The corresponding strains grew at 37°C overnight and then were diluted (1:100) in fresh medium and cultured to an OD_600_ of 0.6. Next, they were induced by 0.1 mM IPTG at 30°C, and OD_600_ values were measured at various times. Controls were set and treated as above, except that IPTG was not added. For the energy toxin experiment, 2,4-dinitrophenol (DNP) with a final concentration of 1 mmol/L was added while IPTG was added. Controls were set and treated as above, except that was not DNP added. Almost all holins could depolarize the cell membrane in the presence of some energy toxin compounds (2,4-DNP or potassium cyanide) and cause membrane damage to form in advance (Heagy, 1950; Anderson and Doermann, 1952). Therefore, the sensitivity to DNP was an important standard to judge holin.

### Morphological Observation of Bacteria

Morphological changes in *E. coli* BL21 (DE3) cells expressing endopeptidase and holin were examined by light microscopy and transmission electron microscopy (TEM). Briefly, DE3: pET21a-endopeptidase and DE3: pET21a-holin were induced to express the target gene, respectively, as described above. Samples were gram-stained and observed by light microscopy. Ultrastructural changes in *E. coli* were visualized after the expression of endopeptidase and holin for 4 h by transmission electron microscopy (TEM; H-7650; Hitachi). The number of abnormal cells in three independent experiments was counted for statistical analysis.

### Measurement of β-Galactosidase Activity

The membrane permeabilization of DE3: pET21a-endopeptidase or DE3: pET21a-holin or their TMD deletion mutants was assessed by measurement of β-galactosidase activity using *O*-nitrophenyl-beta-D-galactopyranoside (ONPG), which is a substrate for the cytoplasmic β-galactosidase enzyme (Zhu et al., 2014). Similarly, the indicated *E. coli* strains were induced as described above, and then 500 μl of extracellular supernatant was taken to mix with 100 UL of 20 mM ONPG. The mixture was incubated at 37°C for 1 h, and Na_2_CO_3_ (0.5 mol/L) was added to stop the reaction. Finally, the supernatant of the mixture after centrifuged at 8,000 g for 10 min was used to measure the optical density at 420 nm (OD_420_). The β-galactosidase activity (U/ml) was calculated as follows: (OD_420_⋅V)/(T⋅VS⋅0.0045), where V is the volume of the mixture (ml); T is the time of reaction (min); VS is the volume of sample that was used to detect OD_420_ (ml); and 0.0045 is the extinction coefficient (ml/nmol).

### Co-expression of Endopeptidase and Holin in *Escherichia coli*

The growth curves of different strains (DE3: pETDuet-1, DE3: pETDuet-endopeptidase, DE3: pETDuet-holin, and DE3: pETDuet-holin-endopeptidase) were measured as described above. To detect the death or survival of the cell, the bacteria were stained with a fluorochrome live/dead dye kit (Mailian Biotechnology, Shanghai, China). Briefly, after being induced by IPTG for 2 h, different strain cells were collected and stained with backlight live/dead staining according to the manufacturer’s instructions. Three microphotographs of each sample were taken under a fluorescence microscope (Leica DMB, Leica, Germany) with an N21 (BP 515-560) filter for green fluorescence and a CY5-T (BP 635/10) filter for red fluorescence. Anti-S-tagged mAb and anti-His-tagged mAb (CST, Trask Lane Danvers, MA, United States) were used as primary antibodies for the detection of endopeptidase and holin, respectively. The number of dead bacteria from three independent experiments was counted and used for statistical analysis.

### Effect of Endopeptidase and Holin on *Synechocystis* sp. PCC 6803

To achieve the expression of endopeptidase and holin in cyanobacteria, the plasmids pHB-endopeptidase and pHB-holin were constructed, respectively, and transformed into *Synechocystis* sp. PCC 6803 as described previously (Gao et al., 2007). Briefly, cyanobacteria cells at the OD_750_ = 0.8 were collected and mixed with the plasmid pHB-endopeptidase and pHB-holin (final concentration: 5 ng/μl) to further incubate at 25°C for 4 h under light conditions. Then the cultures were evenly spread onto the BG11 plate that was initially covered with a piece of the nitrocellulose filter membrane. After 24 h of light culture for the BG11 plate, the filter membrane was transferred to the new BG11 plate containing 10 μg/ml spectinomycin and 50 mmol/L glucose, and then cultured until the recombinant cyanobacteria (PCC6803: pHB-endopeptidase or PCC6803: pHB-holin) appeared on the selective BG11 plate, and then recombinant cyanobacteria were confirmed by PCR. The primers of ORF123-FW/RV and ORF124-FW/RV were used for PCR amplification (Table 2).

To induce the expression of endopeptidase and holin, Cu^2+^ was added at a final concentration of 320 nM to the BG11 liquid medium as the inducing medium. Then the recombinant cyanobacteria cells were adjusted to the initial OD_750_ of 0.13 using the inducing medium, and cultured at 25°C with a light intensity of approximately ∼30 μE⋅m^–2^s^–1^ in an illuminating incubator. After that, the growth of cyanobacteria was monitored over a 5-day time course, and at the indicated time point, samples were taken to measure the OD_750_ value by using a spectrophotometer. Wild-type *Synechocystis* sp. PCC 6803 was included as the control group. Cyanobacteria cells were collected after being cultured for 5 days in the inducing medium for the determination of cyanobacteria cell viability (Tian et al., 2019) and the preparation of samples for TEM observation. Briefly, 100 μl of different cyanobacteria cells (PCC6803WT; PCC6803: pHB-endopeptidase; PCC6803: pHB-holin) should be put into 96-well plate holes, respectively, 10 μl WST-8 reagent was added (Beyotime, Shanghai, China), and then the mixture was incubated with the light on a constant temperature shaking table for 2 h to measure the absorbance of 457 nm.

### Statistical Analysis

The *T*-test by Graphpad Prism (version 8.0) was chosen for the cases. The data were presented as mean ± standard deviation from at least three independent experiments. The statistical significance was defined as a *p*-value of < 0.05.

### GenBank Accession Numbers

The GenBank accession numbers of endopeptidase and holin from cyanophage PaV-LD are ADZ31630.1 and ADZ31631.1, respectively.

## Results

### Molecular Characterization of the Endopeptidase and Holin

Two adjacent genes, ORF123 and ORF124 in the cyanophage Pav-LD genome, were predicted to encode an endopeptidase and a holin, respectively (Supplementary Figure 1A). The ORF123 was 594 bp and encoded a 197-aa protein with a deduced molecular mass of 21.2 kDa. The ORF124 was 363 bp and encoded a 120-aa protein with a deduced molecular mass of 13.8 kDa. The endopeptidase or holin lacking TMD contained 158 and 91 aa, with molecular weights of 16.4 and 9.9 kDa, respectively. The homologous search revealed that the endopeptidase had low homology (<50%) with other known endopeptidases in NCBI databases; however, functional domain analysis revealed that it harbored a conserved peptidase_M23 domain located at 91–186 aa (Supplementary Figure 1B) and a hydrophobic TMD in its N-terminus (Supplementary Figure 1C). The holin had no sequence homology to other proteins and possessed one putative hydrophobic TMD in the N-terminal sequence (Supplementary Figure 1D).

**FIGURE 1:**
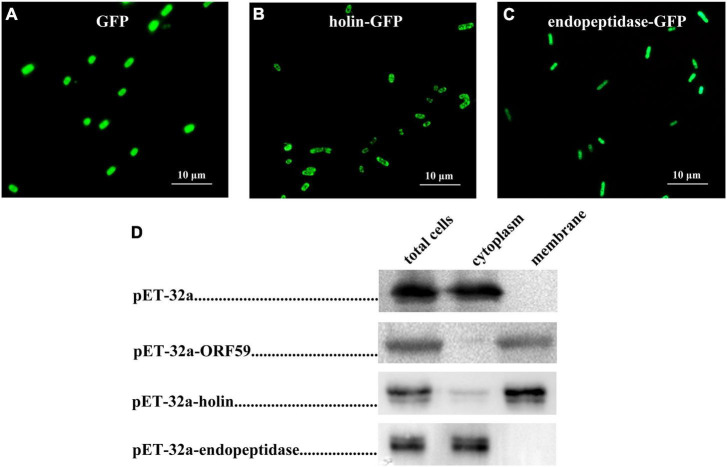
Localization of holin and endopeptidase in *Escherichia coli*. **(A)** Fluorescence image of *E. coli* (DE3: pET32a-GFP) expressing GFP. **(B)** Fluorescence image of *E. coli* (DE3: pET-32a-holin*-*GFP) expressing holin*-*GFP. **(C)** Fluorescence image of *E. coli* (DE3: pET-32a-endopeptidase*-*GFP) expressing endopeptidase*-*GFP. **(D)** The subcellular localization of holin and endopeptidase were analyzed using Western blotting.

To examine the localization of endopeptidase and holin in *E. coli*, these proteins were first expressed by fusion with the GFP tag at its C-terminus (endopeptidase-GFP, holin-GFP), and the GFP fluorescence was examined by fluorescence microscopy to show the distribution of endopeptidase-GFP or holin-GFP. As shown in Figure 1B, green fluorescence was observed in the peripheral region of the holin-GFP expressed cells of *E. coli*, looking like a ring-like appearance. However, in the endopeptidase-GFP expressed *E. coli* cells, green fluorescence seemed to fill the whole cell (Figure 1C). As for the control group, the vector-expressed GFP was distributed in the whole region of *E. coli* (Figure 1A). To further verify the localization of holin or endopeptidase in *E. coli*, the protein was expressed by fusion with His-tag at its C-terminus, then cytoplasm and membrane fractions were, respectively, detected by Western blotting. The results showed that endopeptidase-His existed in the cytoplasm (41.2 kDa; Figure 1D), while holin-His was predominantly in the membrane fraction (33.8 kDa; Figure 1D). All of these results suggested that the holin was a membrane-associated protein, while endopeptidase was more likely to be a cytoplasmic protein.

### Inhibitory Effect of the Endopeptidase and Holin on *Escherichia coli* Growth

**FIGURE 2:**
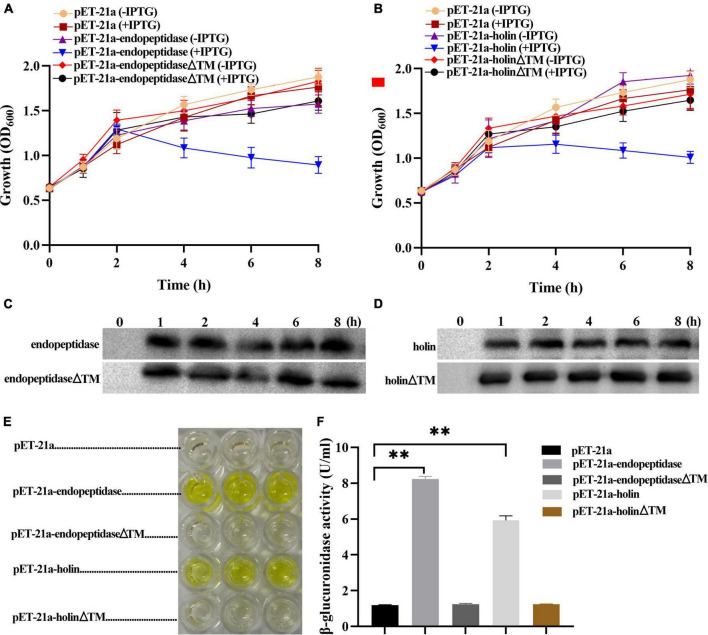
Effect of endopeptidase and holin on the growth activity of *E. coli*. **(A)** The growth curves of induced and uninduced strains (DE3: pET-21a, DE3: pET-21a- endopeptidase, and DE3: pET-21a-endopeptidaseΔTM). **(B)** The growth curves of induced and uninduced strains (DE3: pET-21a, pET-21a-holin, and DE3: pET-21a- holinΔTM). **(C)** Western blotting was performed with total cellular proteins from IPTG-induced DE3: pET-21a-endopeptidase and DE3: pET-21a-endopeptidaseΔTM. **(D)** Western blotting was performed with total cellular proteins from IPTG-induced DE3: pET-21a-holin and DE3: pET-21a-holinΔTM. **(E)** The color of the solution changed after adding galactosidase substrate (ONPG) to the supernatant of different strains (DE3: pET-21a, DE3: pET-21a-endopeptidase, DE3: pET-21a-endopeptidaseΔTM, DE3: pET-21a-holin, and DE3: pET-21a-holinΔTM). **(F)** The β-galactosidase activity was determined for extracellular supernatant from induced strains. The data were shown as means ± standard deviation from three independent experiments. The “^**^” indicates statistical significance (*T*-test, *p* < 0.01).

To probe whether the endopeptidase and holin alone performed their expected functions, we first examined the effect of their expression on the growth of *E. coli.* Growth curve analysis based on OD_600_ value showed that the DE3: pET-21a-endopeptidase or DE3: pET-21a-holin cells grew normally within 2 h after the induction of IPTG compared with the control groups; however, their growth began to go down after 2 h of IPTG induction (Figures 2A,B), revealing that the endopeptidase and holin both have an inhibitory effect on the growth of *E. coli*. Additionally, we also tested the sensitivity of the DE3: pET-21a-holin cells to DNP. The result showed that the addition of DNP brought forward the growth inhibition of holin in *E. coli*, as evidenced by the fact that the DE3: pET-21a-holin cells began to go down only after 1 h of IPTG induction (Supplementary Figure 2B). Therefore, all these results indicated that the endopeptidase or holin alone could cause cell death in its expressed *E. coli* cells, which underlaid their inhibitory phenotype.

A hallmark of viable cells is an intact membrane, and dying cells are usually linked with compromised membranes. To further figure out the mechanism of growth inhibition caused by endopeptidase and holin, we measured the activity of extracellular β-galactosidase to indicate the status of membrane permeability (Supplementary Figure 2A). As expected, the results showed that after adding ONPG to the supernatant of DE3: pET-21a-endopeptidase or DE3: pET-21a-holin cell culture, the color of the solution changed significantly and finally turned yellow compared with the strain DE3: pET-21a (Figure 2E). Meanwhile, the activities of extracellular β-galactosidase in the DE3: pET-21a-endopeptidase or DE3: pET-21a-holin cells were significantly higher than those in control, which the results were shown in Figure 2F (*T*-test, *p* < 0.01), indicating that the endopeptidase or holin had abilities to cause membrane damages.

### Transmembrane Domain of Endopeptidase and Holin Were Essential for Their Bacteriolytic Activity

To elucidate the TMD functions of endopeptidase and holin, the deletion mutants (pET-21a-endopeptidaseΔTM and pET-21a-holinΔTM) were, respectively, constructed. The expression of endopeptidaseΔTM or holinΔTM proteins in *E. coli* BL21 (DE3) was verified by Western blotting (Supplementary Figure 2C), then we monitored the inhibitory effect of the two mutants on bacterial growth and also measured the leakage of intracellular enzymes into the supernatant (Supplementary Figure 2A). The growth curves revealed that both TMD deletions resulted in the loss of growth inhibition of *E. coli* caused by the full-length endopeptidase and holin (Figures 2A,B). Meanwhile, the β-galactosidase activity assay also showed the color of the solution did not change after adding ONPG into the supernatant of DE3: pET-21a-endopeptidaseΔTM or DE3: pET-21a-holinΔTM cell culture compared with the control (Figure 2E). After TMD deletion from endopeptidase or holin, the amount of β-galactosidase in the supernatant of *E. coli* cells decreased sharply compared with wild strains (DE3: pET-21a-endopeptidase or DE3: pET-21a-holin; T-test, *p* < 0.01; Figure 2F). Taken together, these data indicated that TMDs of endopeptidase or holin were essential for their bacteriolytic activity.

### Endopeptidase and Holin Exerted Bacteriolytic Activity by Causing Membrane Damage

**FIGURE 3:**
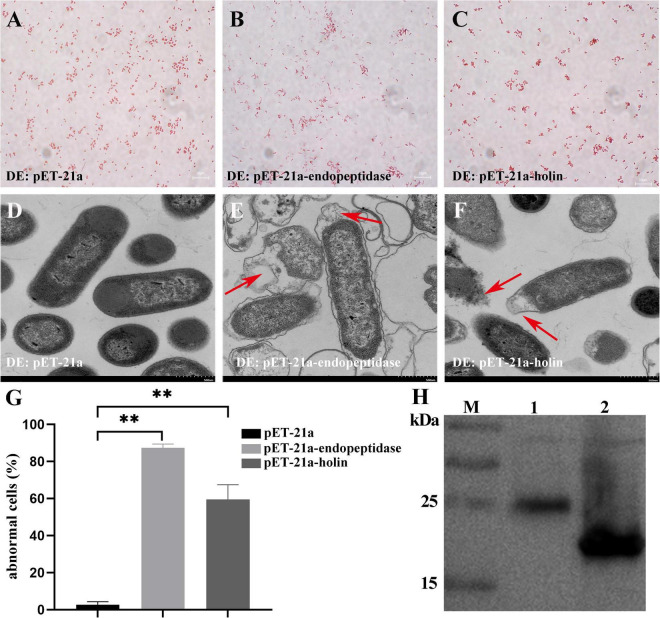
Effect of endopeptidase and holin on *E. coli* cell morphology. **(A)** Gram staining of induced DE3: pET-21a. **(B)** Gram staining of induced DE3: pET-21a-endopeptidase. **(C)** Gram staining of induced DE3: pET-21a-holin. **(D)** Induced DE3: pET-21a under TEM. **(E)** Induced DE3: pET-21a-endopeptidase under TEM. **(F)** Induced DE3: pET-21a-holin under TEM. Red arrows in panels **(E,F)** indicate morphological changes. **(G)** Percentage of abnormal cells of different *E. coli* strains (DE3: pET-21a, DE3: pET-21a-endopeptidase, and pET-21a-holin). Each column represents the mean of three independent experiments, and error bars indicate the standard deviation. The “^**^” indicates statistical significance (T-test, *p* < 0. 01). **(H)** The expression of endopeptidase and holin proteins was identified using Western blotting. M: protein marker; lane 1: a fusion endopeptidase protein ∼25 kDa; lane 2: a fusion holin protein ∼18 kDa.

To decipher the mechanism of growth inhibition in *E. coli*, after determining the expression of endopeptidase or holin (Figure 3H), we further observed the morphological changes caused by the overexpression of endopeptidase or holin by gram staining and TEM. These results revealed that the ultrastructural morphology of some *E. coli* that expressed the endopeptidase was abnormal, which were characterized by the rupture of the cell wall and cell membrane, loose cell structure, overflow of cell content, loss of cell content, and condensation of cell content (Figures 3B,E). Similarly, *E. coli* cells expressing the holin exhibited a blurred cellular boundary, slightly shrunk between cell wall and membrane, and a lighter cell color (Figures 3C,F). In contrast, the control cells (DE: pET-21a) showed normal morphological structure, including intact cell membrane, clear cell wall, and dense cellular contents (Figures 3A,D). In addition, we could see more abnormal cells in Supplementary Figures 3B,C, and the results of statistical analysis showed that the abnormal cell rate of DE3: pET-21a-endopeptidase or DE3: pET-21a-holin increased significantly, up to 87 and 59%, respectively, compared with the control cells (T-test, *p* < 0.01; Figure 3G). Together with the above results, we conclude that the endopeptidase or holin from the PaV-LD both harbors bacteriolytic activity by disrupting the membrane integrity of *E. coli*.

### Synergistic Effect of the Endopeptidase and Holin on Bactericidal Activity

**FIGURE 4:**
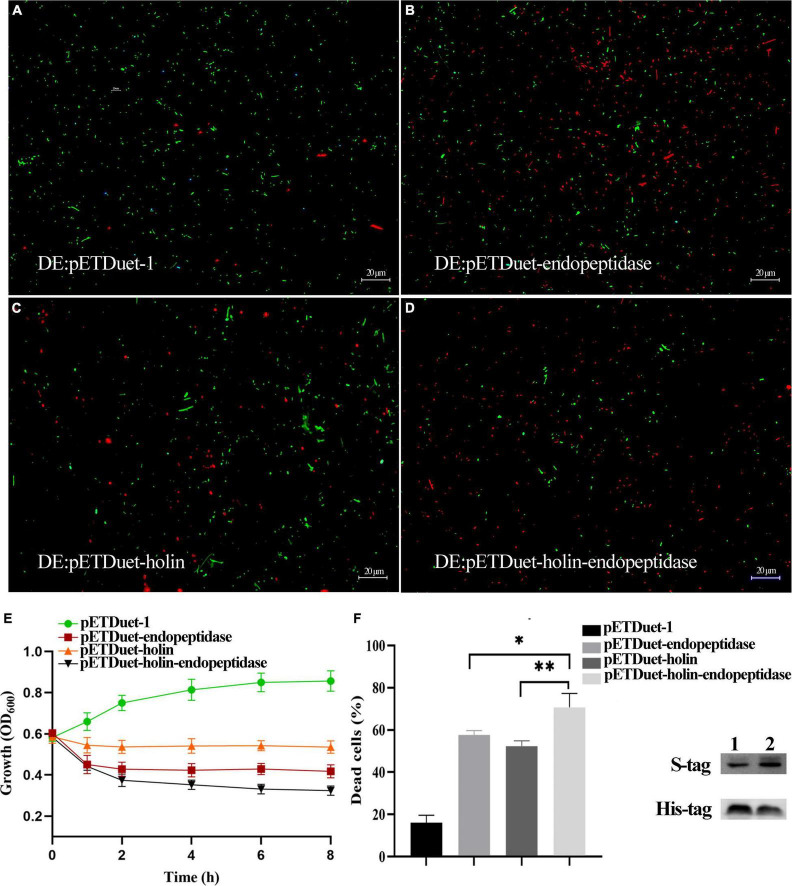
Co-expression of endopeptidase and holin in *E. coli* BL21 (DE3). **(A)** Fluorescent staining of induced DE3: pETDuet-1. **(B)** Fluorescent staining of induced DE3: pETDuet-endopeptidase. **(C)** Fluorescent staining of induced DE3: pETDuet-holin. **(D)** Fluorescent staining of induced pETDuet-holin-endopeptidase. The cells with green fluorescence represent living bacterial cells, and those with red fluorescence represent dead cells. **(E)** The growth curves of induced different *E. coli* strains. **(F)** The percentage of dead cells of different *E. coli* strains. Lane 1: Western blotting was performed using total cellular proteins from IPTG-induced DE: pETDuet-endopeptidase or DE: pETDuet-holin. Lane 2: Western blotting was performed with total cellular proteins from IPTG-induced DE: pETDuet-holin-endopeptidase. The data were shown as means ± standard deviation from three independent experiments. The “*” (T-test, *p* < 0. 05) and “^**^” indicates statistical significance (T-test, *p* < 0. 01).

To further explore whether PaV-LD also used the efficient “holin-endolysin” two-component cleavage system to lyse bacteria, endopeptidase and holin were co-expressed in *E. coli* BL21(DE3). The growth curve results showed that the growth of *E. coli* (DE3: pETDuet-holin-endopeptidase) was inhibited severely than that of *E. coli* DE3: pETDuet-endopeptidase or DE3: pETDuet-holin (Figure 4E). Meanwhile, the live/dead dye assays revealed that both DE3: pETDuet-holin (T-test, *p* < 0.01) and DE3: pETDuet-endopeptidase (T-test, *p* < 0.05) had significantly different mortality rates with DE3: pETDuet-holin-endopeptidase (Figures 4A–D). In particular, the co-expression of the holin and endopeptidase resulted in nearly 68% cell death (red staining), while the lethal rates of endopeptidase or holin alone to *E. coli* was 58 and 52%, respectively (Figure 4F). These results suggested that endopeptidase and holin had synergistic functions in the bactericidal activity of *E. coli*.

### Effect of Endopeptidase and Holin on *Synechocystis* sp. PCC 6803

**FIGURE 5:**
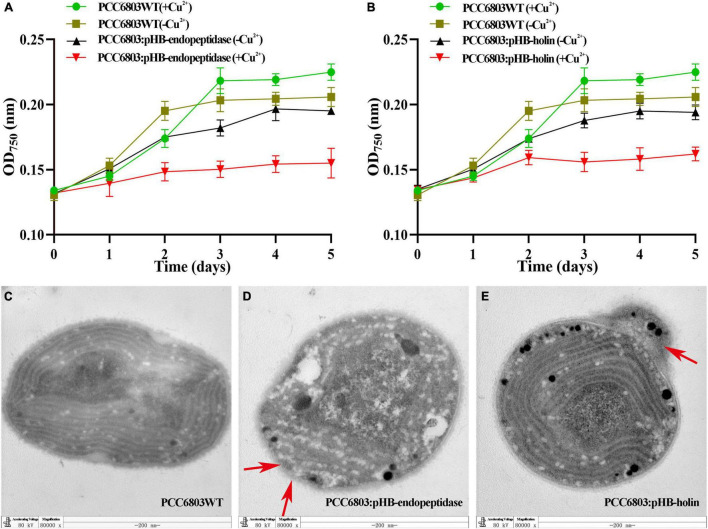
Growth curves and transmission electron microscopy (TEM) observation of recombinant cyanobacteria. **(A)** The growth curves of induced and uninduced strains (PCC6803WT and PCC6803: pHB-endopeptidase). **(B)** The growth curves of induced and uninduced strains (PCC6803WT and PCC6803: pHB-holin). The data were shown as means ± standard deviation from three independent experiments. **(C)** Induced PCC6803WT under TEM. **(D)** Induced PCC6803: pHB-endopeptidase under TEM. **(E)** Induced PCC6803: pHB-holin under TEM. Red arrows in panels **(D,E)** indicate morphological changes.

To further verify the functions of endopeptidase and holin in cyanobacteria cells, the cyanobacterium PCC6803 was selected as host cells to express the endopeptidase or holin, and the growth and ultrastructural morphology of PCC6803 were examined. First, we verified by PCR that the target gene was indeed in the genome of recombinant cyanobacteria (Supplementary Figures 4A,B). Growth curve analysis revealed that the recombinant cyanobacteria PCC6803: pHB-endopeptidase displayed a slower growth rate than the wild type upon induction in the presence of Cu^2+^. Although the PCC6803: pHB-endopeptidase or PCC6803WT had indistinguishable growth rates in the absence of Cu^2+^, they both exhibited higher growth rates than the PCC6803: pHB-endopeptidase in the presence of Cu^2+^ (Figure 5A). The data indicated that endopeptidase led to the slow-growth phenotype. Similarly, the expression of holin also produced a comparative result in the PCC6803 (Figure 5B). The ultrastructural morphology of PCC6803WT, PCC6803: pHB-endopeptidase, and PCC6803: pHB-holin after induction of Cu^2+^ were observed, showing that the ultrastructure of wild-type PCC6803 was normal and intact in each part (Figure 5C). However, the PCC6803 cell expressing the endopeptidase or holin displayed obvious abnormal morphology, such as disrupted protoplasts, blurred or disappeared cell walls, and cell content leakage (Figures 5D,E). In addition, more abnormal cells were shown in Supplementary Figures 2E,F. Moreover, when the endopeptidase and holin were expressed, the absorbance values of PCC6803 at 457 nm were much lower than that of wild type (*T*-test, *p* < 0.01), which indicated that the expression of the endopeptidase or holin alone could reduce the cell viability of PCC6803 (Supplementary Figure 4C). Overall, these findings suggest that the endopeptidase or holin from the cyanophage PaV-LD exhibited bacteriolytic activity against not only bacterial species but also cyanobacteria cells.

## Discussion

The functions of endopeptidases and holins derived from many phages have been extensively studied (Bavda and Jain, 2020; Głowacka-Rutkowska et al., 2020; Zampara et al., 2020). However, few studies focused on their homologs in cyanophages. In this study, the endopeptidase and holin encoded by a *P. agardhii* cyanophage PaV-LD were investigated for their roles in causing cell death of *E. coli* and cyanobacteria cells.

Endopeptidases act on the peptide crosslinking the stem structure of peptidoglycans and also contain many distinct groups, such as the M23 peptidase family (Rawlings et al., 2016). Over the past years, many endopeptidases from different bacteriophages have been identified and characterized to be members of the M23 family, and they function as zinc metallopeptidases with a range of specificities. Furthermore, many proteins containing the M23 peptidase domain have specific hydrolytic activity on peptidoglycan, resulting in bacteriolytic phenotype (Schindler and Schuhardt, 1964; Thumm and Gtz, 2010). In this study, although the endopeptidase from the cyanophage PaV-LD had no high similarity with other known endopeptidases in the database that were derived from bacteriophages, it harbored an M23 peptidase domain, indicating that it might have a bacteriolytic function. Previously, the growth inhibition of *Synechocystis* sp. PCC6803 by co-expression of the lytic system (endopeptidase and holin) also suggested the probable role (Li et al., 2013).

Under these circumstances, we expressed the endopeptidase in the *E. coli* expression system to investigate whether it acted alone to exert bacteriolytic function. As expected, our data demonstrated that the endopeptidase alone can cause the lytic phenotype of *E. coli* and cyanobacteria based on different experiments, including observation of growth inhibition, membrane damage, and leakage of intracellular enzymes. It is generally recognized that some endolysins without signal peptides exert their enzyme activities after being transported through the inner membrane pores formed by holin (White et al., 2011). However, recent reports also showed some endolysins from bacteriophages still displayed activity in the absence of holin (Min et al., 2004; Xu et al., 2005).

Holins are small hydrophobic transmembrane proteins encoded by phages with low homology and diverse structures (Saier et al., 2015). In our study, ORF124, encoding the putative holin of the cyanophage PaV-LD, was found in the lysis module. Bioinformatic analysis showed that the holin from PaV-LD consisted of one putative hydrophobic TMD and might belong to Class III holins. In this study, biological evidence for the holin-like character of ORF124 was obtained using *E. coli* and cyanobacteria. The expression of the holin from PaV-LD caused shrinkage of the cellular membrane, the disruption and damage of the cell membranes in *E. coli*, which was consistent with the previous studies (Song et al., 2016; Nl et al., 2020). Additionally, the holin from PaV-LD was classical and sensitive to DNP, indicating that ORF124 shows the same membrane energy sensitivity as other holins (Díaz et al., 1996; Martín et al., 1998; Grundling et al., 2001). Previous studies have shown that holin plays a crucial role in assisting endolysin dissolve the host cell wall (To and Young, 2014). In this study, to further investigate the physiological role of ORF124 during the process of endopeptidase release, we co-expressed the holin and endopeptidase in *E. coli*. The co-existence of holin and endopeptidase had an obvious bactericidal effect on *E. coli* than that of holin or endopeptidase alone, which indicated that holin might help the release of endopeptidase. Similar phenomena have been reported in other phages, including the lytic systems of bacteriophage phiSASD1 and OP2-Like Phage X2 (Nl et al., 2020; Wu et al., 2021).

The TMDs are major components of holins and play important roles in their active functions. Previous studies have shown that both TMD1 and TMD2 of phage HolGH15 are indispensable for the function of the full-length protein (Song et al., 2016). In our study, the expression of endopeptidaseΔTM or holinΔTM in *E. coli* was similar to that of the full-length protein (Figures 2C,D). However, the expression of endopeptidasΔTM or holinΔTM from PaV-LD could not inhibit *E. coli*. Besides, the same biological evidence was obtained using the extracellular β-galactosidase activity experiment. Therefore, TMD was an important part of holin and endopeptidase of PaV-LD, and the lack of TMD would lead to the loss of its biological activity. Meanwhile, we also proved that the TMD of endopeptidase from the PaV-LD was essential for its bacteriolytic activity.

*Planktothrix agardhii* HAB0637 is the natural host of the cyanophage PaV-LD (Gao et al., 2012), and it will elucidate the lytic mechanism of PaV-LD if functions of the endopeptidase and holin were verified in the natural host cyanobacteria. Unfortunately, HAB0637 is not naturally transformable and lacks a reliable expression vector so far (Li et al., 2013). *Synechocystis* sp. PCC6803 is one of the most studied cyanobacteria and is used in the biotechnology field widely since it cannot only be easily transformed but also has a copper ion-induced expression platform controlled by a *petE* promoter (Gao et al., 2007). For that reason, we chose *Synechocystis* sp. PCC6803 cells to verify the individual function of endopeptidase and holin of Pav-LD in this study. Expectedly, the endopeptidase or holin alone destroyed the cell wall or membrane, resulting in significant inhibition of cell growth and a reduction of cell activity that is similar to the function of endopeptidase and holin in the *E. coli* system. Taken together, these data confirmed that the endopeptidase or holin from the PaV-LD has bacteriolytic activity on cyanobacteria cells.

In this study, endopeptidase and holin derived from *P. agardhii* cyanophage PaV-LD were identified and characterized. Both endopeptidase and holin had a transmembrane domain at the N-terminal without a signal peptide. The expression of endopeptidase or holin in *E. coli* cells could inhibit the growth of host cells and cause morphological changes in *E. coli* cells. Meanwhile, the transmembrane domains of endopeptidase and holin were essential for the activity of the full-length protein. Furthermore, the two proteins displayed a synergistic effect when the endopeptidase and holin were co-expressed in *E. coli*. We confirmed that both endopeptidase and holin could inhibit the growth of *Synechocystis* sp. PCC6803. Taken together, our study proved that endopeptidase or holin alone caused the damage or dissolution of the bacterial cell wall and cell membrane, and the combined effect of the two was better.

## Data Availability Statement

The original contributions presented in the study are included in the article/Supplementary Material, further inquiries can be directed to the corresponding author.

## Author Contributions

L-HM and Q-YZ designed and conducted the experiments. L-HM analyzed the data, evaluated the results, and drafted the manuscript. FK and ZZ advised in the research work, interpretation of data, and helped in drafting and writing of the manuscript. All authors approved the manuscript.

## Conflict of Interest

The authors declare that the research was conducted in the absence of any commercial or financial relationships that could be construed as a potential conflict of interest.

## Publisher’s Note

All claims expressed in this article are solely those of the authors and do not necessarily represent those of their affiliated organizations, or those of the publisher, the editors and the reviewers. Any product that may be evaluated in this article, or claim that may be made by its manufacturer, is not guaranteed or endorsed by the publisher.

## Footnotes

1
http://www.cbs.dtu.dk/services/TMHMM/
