# Supplementary material for: Functional Analysis of the Endopeptidase and Holin From Planktothrix agardhii Cyanophage PaV-LD
Source: Front Microbiol. 2022 Apr 28;13:849492. doi: 10.3389/fmicb.2022.849492 (PMC9096620; doi:10.3389/fmicb.2022.849492)
Supplement: Supplementary file 1 [file Table_1.DOCX]

**Supplementary Material**


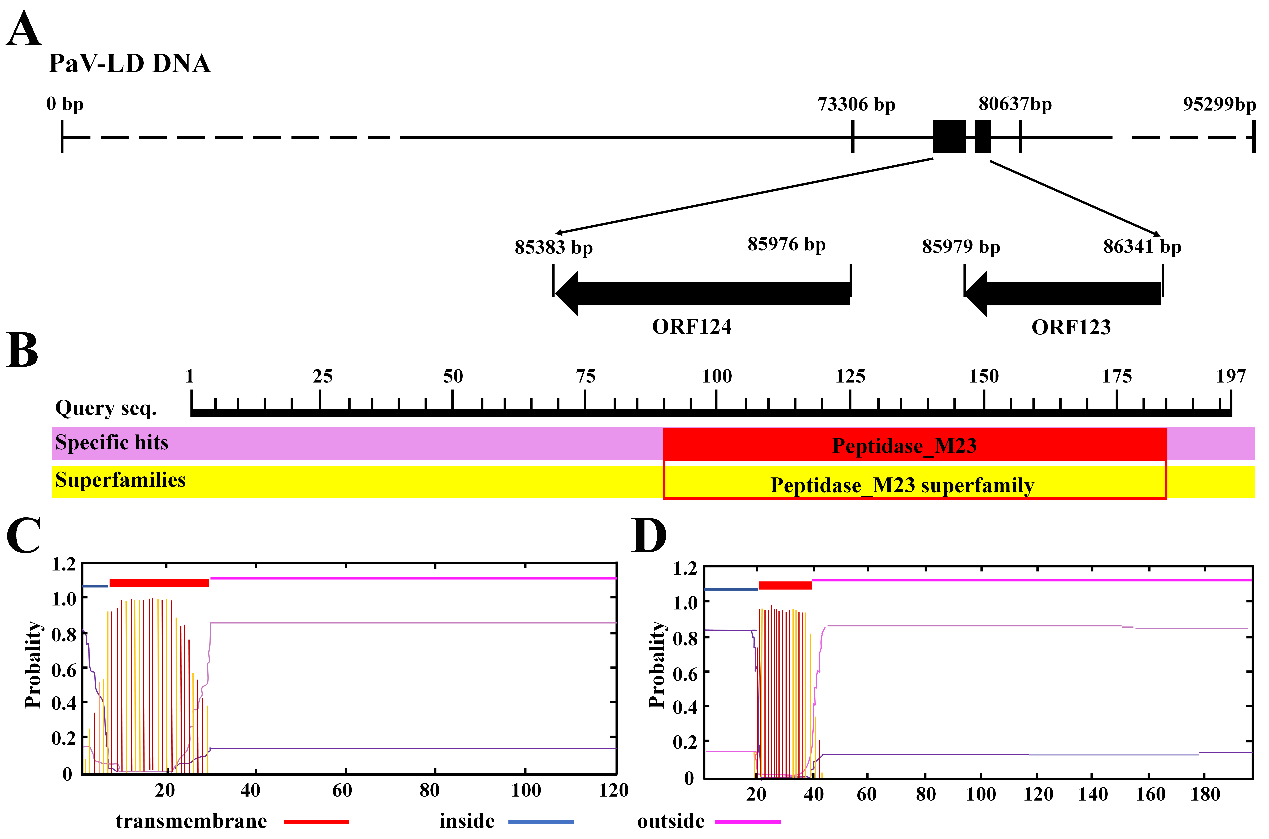


**Fig. S1:** Analysis of ORF123 and ORF124 from the cyanophage PaV-LD genome. (A) Location of ORF123 and ORF124. These two genes were likely to compose the putative lysis module of cyanophage PaV-LD. (B) Putative conserved domain present in the endopeptidase. (C) Prediction of transmembrane segments in endopeptidase. (D) Prediction of transmembrane segments in the holin.


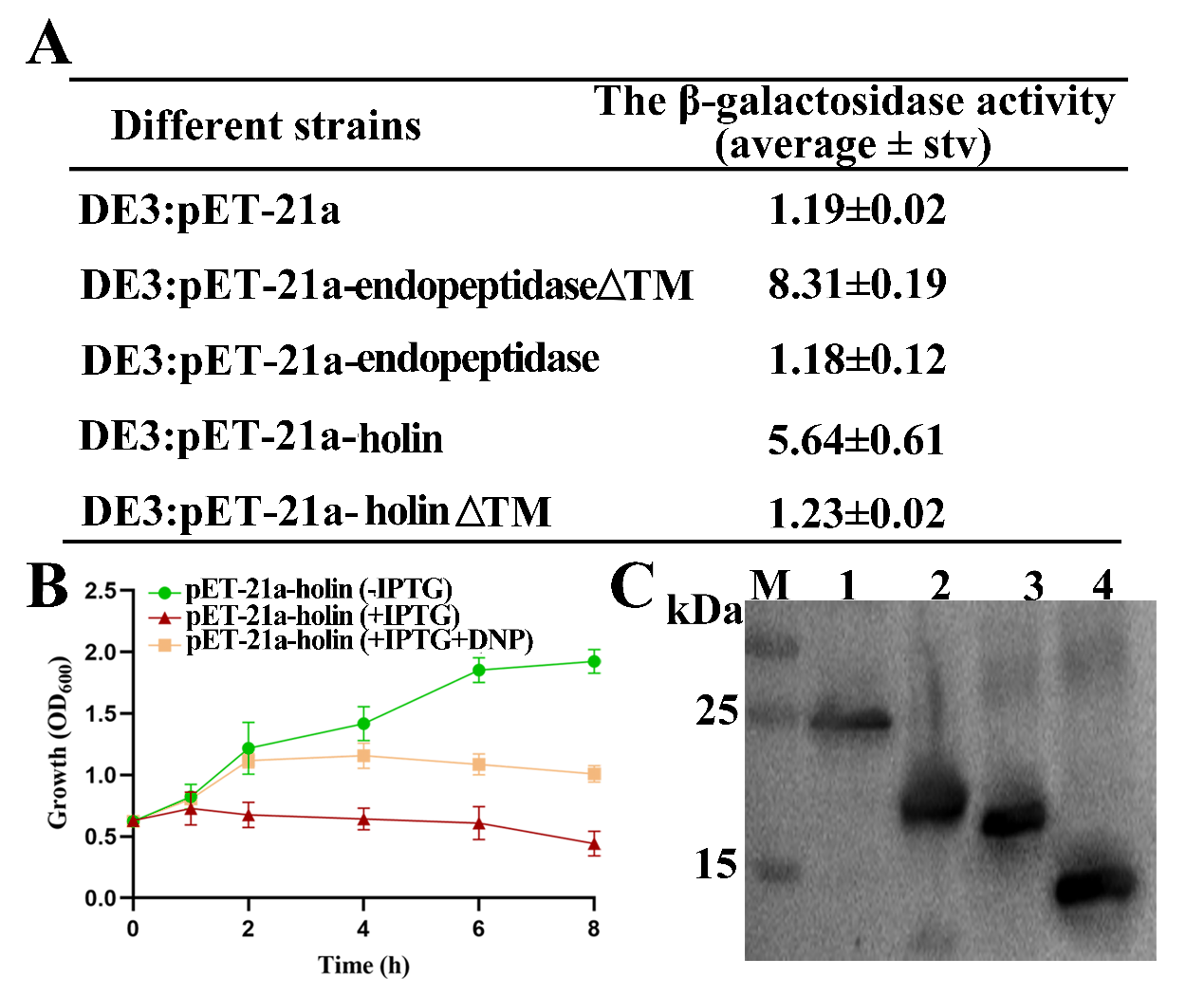


**Fig. S2:** (A) The β-galactosidase activity was determined for extracellular supernatant from induced strains (DE3: pET-21a, DE3: pET-21a-endopeptidase, DE3: pET-21a- endopeptidaseΔTM, DE3: pET-21a-holin and DE3: pET-21a-holinΔTM). (B) Effect of 2,4-dinitrophenol (DNP) on the growth of DE3: pET-21a-holin. The bacterial cultures were induced with IPTG. Uninduced DE3: pET-21a-holin and induced DE3: pET-21a-holin without DNP as controls. (C) The expression of fusion proteins was identified by Western blot. M: protein marker; lan1: a fusion endopeptidase protein ∼25 kDa; lan2: a fusion endopeptidaseΔTM protein ∼20 kDa; lan3: a fusion holin protein ∼18kDa; lan4: a fusion holinΔTM protein ∼14kDa. The data were shown as means ± standard deviation from three independent experiments.


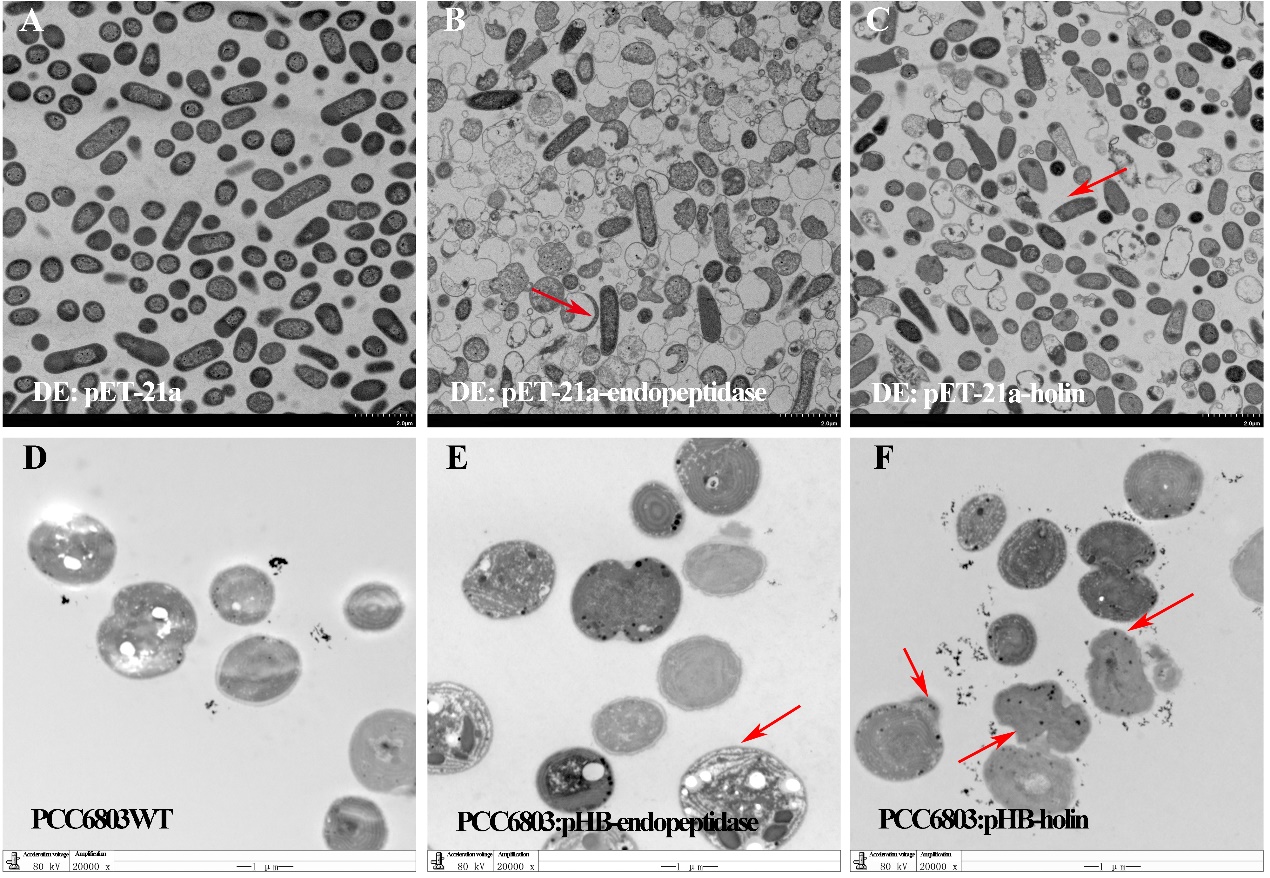


**Fig. S3** (A) Induced DE3: pET-21a under TEM. (B) Induced DE3: pET-21a- endopeptidase under TEM. (C) Induced DE3: pET-21a-holin under TEM. (D) Induced PCC6803WT under TEM. (E) Induced PCC6803: pHB-endopeptidase under TEM. (F) Induced PCC6803: pHB-holin under TEM. Red arrows indicate morphological changes. Red arrows in panels B, C, E and F indicate morphological changes.


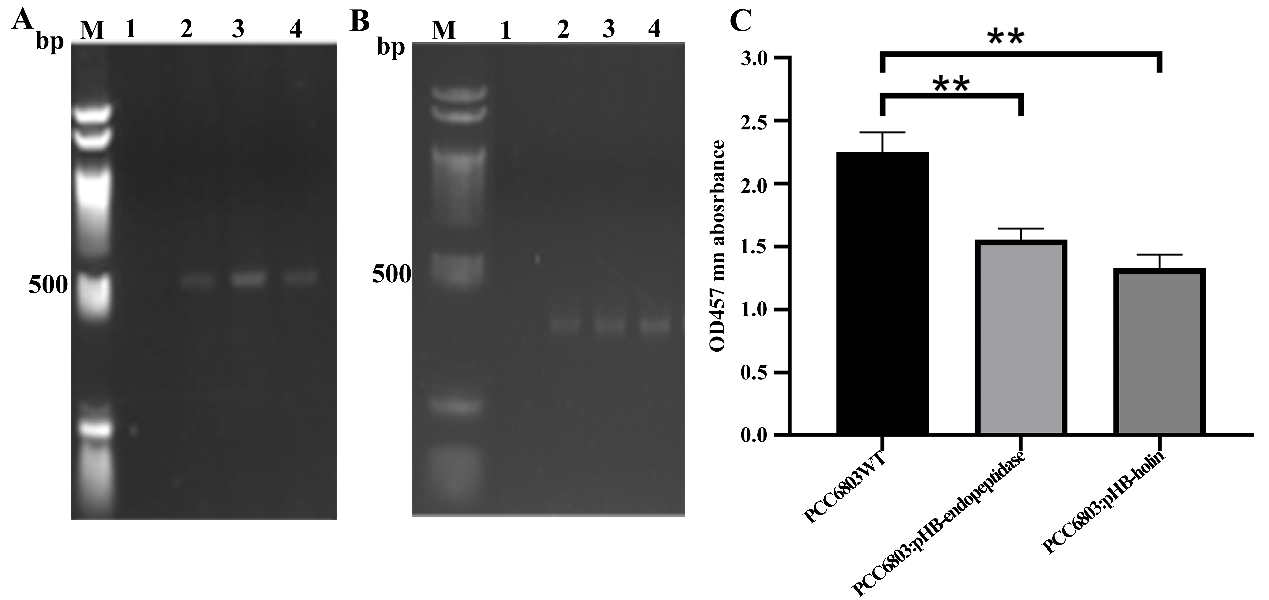


**Fig. S4:** (A) PCR detection of recombinant cyanobacteria PCC6803: pHB-endopeptidase. M: DNA marker; lan1: PCC6803WT; lan2, 3, 4: PCC6803: pHB-endopeptidase (594bp). (B) PCR detection of recombinant cyanobacteria PCC6803: pHB-holin. lan1: PCC6803WT; lan2, 3, 4: PCC6803: pHB-holin (363bp). (C) The cell viability of different induced algal strains (PCC6803WT, PCC6803: pHB- endopeptidase and PCC6803: pHB-holin) were measured by WST-8 assay. The data were shown as means ± standard deviation from three independent experiments. The “**” indicates statistical significance (T-test, *p* < 0. 01).
